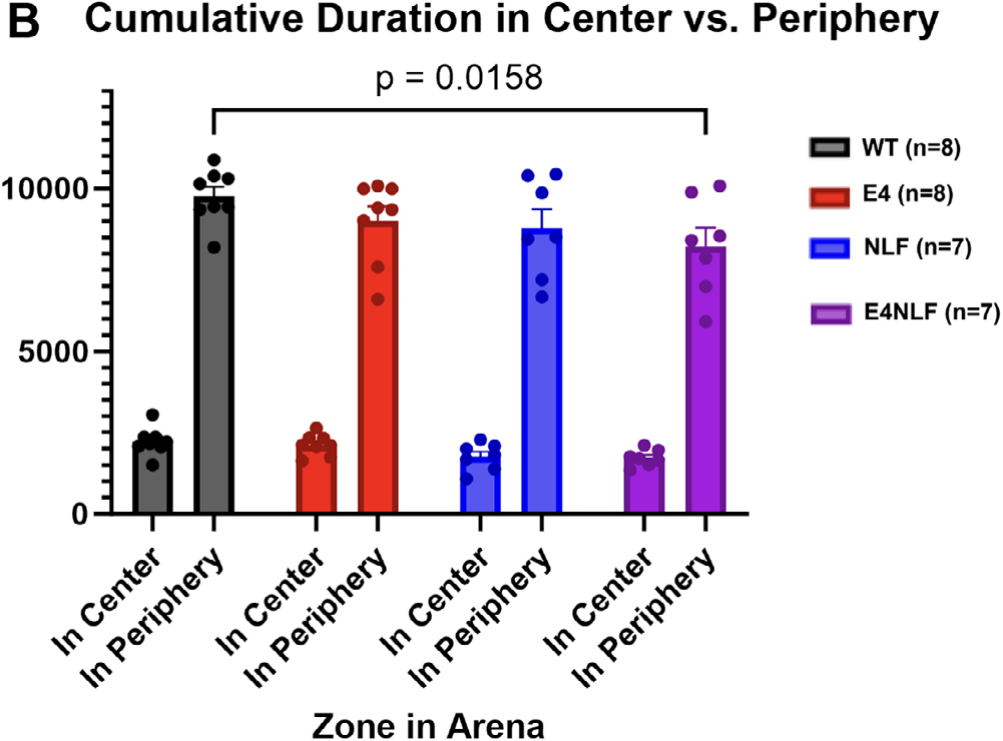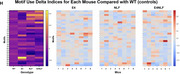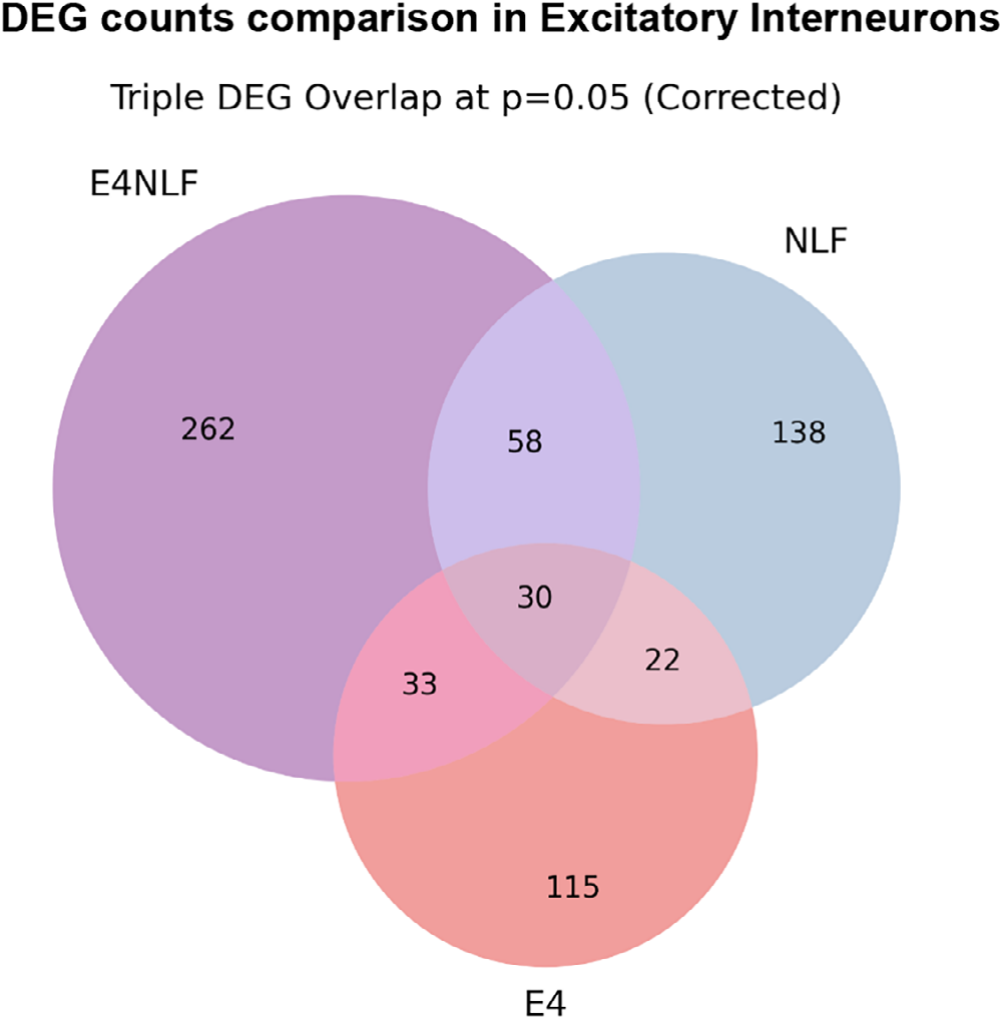# Understanding the genetic interplay between human ApoE4 and novel *App* ^NL‐F^ knock‐in in Alzheimer's Disease male mouse model

**DOI:** 10.1002/alz70855_100104

**Published:** 2025-12-23

**Authors:** Jia Shin, Erica Brady, Stephanie R Miller, Pranav Nambiar, Jorge J Palop

**Affiliations:** ^1^ Gladstone Institute of Neurological Disease, University of California San Francisco, San Francisco, CA, USA; ^2^ University of California, San Francisco, San Francisco, CA, USA

## Abstract

**Background:**

Humanized ApoE4, a major genetic risk factor for late‐onset Alzheimer's Disease (AD), is associated with synaptic dysfunction, neuroinflammation, and deficits in Aβ clearance. To investigate genetic drivers of AD, we used the novel *App* ^NL‐F^ knock‐in mouse model, which incorporates Swedish (NL) and Iberian (F) mutations in the amyloid precursor protein (*App*) gene under its endogenous promoter, replicating human Aβ pathophysiology while preserving native gene regulation. This study examines the behavioral, molecular, and histopathological effects of combining *App* ^NL‐F^ mutations with humanized ApoE4, addressing challenges in modeling human ApoE biology and advancing translational insights into AD pathogenesis.

**Method:**

Four genotypes—ApoE4/*App* ^NL‐F^ (E4NLF), ApoE4 (E4), *App* ^NL‐F^ (NLF), and wild‐type (WT)—were generated using knock‐in technology. Genotypes without humanized ApoE4 retained mouse ApoE. Male mice (15 months old, n = 30) underwent open field behavioral testing analyzed via machine learning pipelines (DeepLabCut, VAME) to detect behavioral alterations. Immunohistochemistry quantified Aβ plaques (Thioflavin‐S, 82e1) and microglial reactivity (Iba1). Soluble Aβ42 and Aβ40 levels were measured via MSD ELISA. Single‐nuclei RNA sequencing (snRNA‐seq) identified differentially expressed genes (DEGs) in hippocampal cell types.

**Results:**

E4NLF mice showed hypoactivity (43% reduction in total distance traveled vs. WT), impaired habituation (*p* < 0.05), and increased slow movements. Aβ plaques were present in both NLF and E4NLF mice, but E4NLF displayed 55% fewer hippocampal plaques (*p* < 0.01). Soluble Aβ42/40 ratios were higher in NLF than E4NLF (*p* < 0.05). Microglia in both genotypes showed amoeboid morphologies near plaques, indicating activation. DEG analysis revealed reductions in Ttr expression (*p* < 0.05) in E4NLF astrocytes and microglia, implicating disrupted Aβ clearance. Excitatory neurons exhibited the highest number of DEGs across all cell types in E4NLF vs. WT and NLF vs. WT.

**Conclusion:**

Combining ApoE4 and *App* ^NL‐F^ mutations produces distinct phenotypes, including hypoactivity, Aβ plaque burden, heightened neuroinflammation, and impaired glial function. Reduced Aβ plaques in E4NLF compared to NLF mice highlight species‐specific differences in Aβ clearance, underscoring functional differences between mouse ApoE and human ApoE4 isoforms. These findings emphasize the limitations of mouse ApoE in accurately modeling human ApoE biology and reinforce the necessity of humanized models to investigate ApoE isoform‐specific roles in AD.